# Genetic Epidemiology of Attention Deficit Hyperactivity Disorder (ADHD Index) in Adults

**DOI:** 10.1371/journal.pone.0010621

**Published:** 2010-05-12

**Authors:** Dorret I. Boomsma, Viatcheslav Saviouk, Jouke-Jan Hottenga, Marijn A. Distel, Marleen H. M. de Moor, Jacqueline M. Vink, Lot M. Geels, Jenny H. D. A. van Beek, Meike Bartels, Eco J. C. de Geus, Gonneke Willemsen

**Affiliations:** Department of Biological Psychology, Vrije Universiteit Amsterdam, Amsterdam, The Netherlands; Leiden University Medical Center, Netherlands

## Abstract

**Context:**

In contrast to the large number of studies in children, there is little information on the contribution of genetic factors to Attention Deficit Hyperactivity Disorder (ADHD) in adults.

**Objective:**

To estimate the heritability of ADHD in adults as assessed by the ADHD index scored from the CAARS (Conners' Adult ADHD Rating Scales).

**Design:**

Phenotype data from over 12,000 adults (twins, siblings and parents) registered with the Netherlands Twin Register were analyzed using genetic structural equation modeling.

**Main outcome measures:**

Heritability estimates for ADHD from the twin-family study.

**Results:**

Heritability of ADHD in adults is estimated around 30% in men and women. There is some evidence for assortative mating. All familial transmission is explained by genetic inheritance, there is no support for the hypothesis that cultural transmission from parents to offspring is important.

**Conclusion:**

Heritability for ADHD features in adults is present, but is substantially lower than it is in children.

## Introduction

Attention Deficit Hyperactivity Disorder (ADHD) is a common neurobehavioral disorder that is characterized by inattention, hyperactivity and impulsivity. It is associated with considerable social, family, behavioral and cognitive dysfunction and is comorbid with depression, bipolar disorder, anxiety, and substance use [1]. ADHD in adults is often left untreated and significantly correlates with previous married status, unemployment, and European ancestry [2]. Genetic studies (family, twin, and adoption studies) demonstrated a strong genetic component in the etiology of the disorder in children [3]–[5], but substantially less is known about the heritability of ADHD in adults. This may partly be due to the fact that some of the earlier work suggested that ADHD is rare in adulthood. Nevertheless, a meta-analysis of follow-up studies on ADHD showed that although syndromatic persistence (i.e., the maintenance of full diagnostic status) is low (around 15%), symptomatic persistence (i.e., the maintenance of partial diagnostic status with impairment) is much higher with a persistence rate of 40–60% [6]. In a population screen of nearly 1000 adults, Faraone *et al.* estimated a prevalence of 2.9% for the narrow and 16.4% for the broad definitions of the ADHD phenotype [7]. Other prevalence estimates vary from study to study and are dependent on the definition and assessment of the phenotype (e.g. self-report or interview data), the gender composition of the sample and the age of the subjects [3], [8], [9]. In a meta-analysis of prevalence studies that followed the DSM-IV criteria for defining the ADHD phenotype, the pooled prevalence was estimated to be 2.5% (95% confidence interval (CI) 2.1–3.1%) [9]. It was noted that out of twelve reviewed studies ten have questioned the validity of DSM-IV criteria when applied to adults. Alternative phenotypic definitions led to a significant variation in prevalence of ADHD (between 2.5 and 42.3%) [9]. The meta-analysis also indicated that the prevalence of adult ADHD has a negative association with age, and that this association is modified by the gender composition of the sample.

A recent study observed strong familial clustering of ADHD in first-degree relatives of adults with ADHD. Compared to a control group adults with ADHD had significantly more first-degree relatives with ADHD (28% versus 5%) [10]. Children born to parents with ADHD have a seven-fold increased risk for developing the disorder than children born to non-affected parents [11]. One twin study looked at the etiological influences on Attention Problems in young adults, as assessed by the Adult Self Report [12]. The contribution of genetic influences was assessed at three points in time [13]. The mean ages of the participants were 19.6, 21.3, and 22.8 years at waves 1, 2, and 3, respectively. At each age the heritability of Attention Problems was estimated around 40%. The stability in attention problems was mainly due to genetic factors [13].

In this manuscript we present a quantitative genetic study of adult ADHD in a large sample of twin families who are registered with the Netherlands Twin Register [14]. Data were collected in 12,594 adult twins (average age 31 years), their siblings (average age 37 years) and parents (average age 55 years). ADHD was assessed with the CAARS (Conners' Adult ADHD Rating Scales, Technical Manual) [15], which is a self-report measure suitable for use in large-scale epidemiological studies. Adults with ADHD are the best informants about their symptoms [16] and the self-report form of the CAARS seems to be an appropriate survey method to evaluate ADHD symptoms in a large family-based cohort. The ADHD index was chosen as a phenotype definition due to its ability to distinguish the ADHD adults from the non-clinical cases (a T-score greater than 65) and the simplicity of the survey administration (12 items on the questionnaire) [16].

Heritability was estimated using data from twins, their parents and siblings with structural equation modeling and under different models of familial resemblance. Adding data from siblings to the classical twin design results in an increase in power to detect non-additive genetic effects [17]. The effects of assortative mating, i.e. the fact that spouses are more similar for a trait or disorder than expected under random assortment, can be detected and accounted for by including data from parents of twins. A small degree of assortative mating is often found for psychiatric disorders [18], [19] in population-based samples. However, resemblance between spouses for ADHD symptoms has not yet been reported. When there is spouse resemblance it is necessary to include its effects in a genetic model. Under random mating genetic effects are uncorrelated in parents. Under non-random assortment genetic effects in parents are correlated and the genetic correlations among the first-degree relatives are elevated that may lead to a biased estimate of heritability if this effect is not taken into account [20].

The classical twin design can inform on the influence of shared environment, but is not informative with respect to how much of the environment that is shared among offspring is transmitted from parents to offspring (cultural transmission). It is easily conceivable that growing up with a parent who scores high on a number of ADHD features influences the phenotype of their offspring. By adding phenotypic data from parents to the classical twin design, vertical cultural transmission, reflecting the non-genetic influence of the parents' ADHD features on their offspring, can be examined. Because ADHD is expected to have a heritable component, vertical cultural transmission will induce genotype-environment correlation [20].

## Methods

### Participants and procedure

Phenotype data were collected in participants from the Netherlands Twin Register (NTR) by survey in 2004–2005 (survey 7) and in an ongoing study (survey 8) that started in 2009. Data collection for survey 7 has been detailed in Distel et al [21]. Briefly, twins and their family members were invited by mail to complete a survey on health, lifestyle, personality and psychopathology. A group of 200 unrelated subjects completed the questionnaire the second time after 6-months to obtain retest data. For survey 8, participants could complete the survey online or by paper and pencil. Individuals were invited by letter, which contained a personalized login-name and password to complete the survey online. If the online survey was not completed within 6 weeks, a reminder was sent together with a paper copy of the survey that could be mailed back free of charge. For this study we analyzed the ADHD index data from survey 7 and added data from survey 8 that were available from the (ongoing) online data collection. For 2,519 subjects repeated-measures (i.e. survey 7 and survey 8 internet data) were available from both time points. If two scores were available, the first one (survey 7) was chosen. In total, there were phenotype data available for 15,273 individuals. We excluded offspring of twins (N = 341), subjects with incomplete data on sex (N = 190), who were younger than 18 years or who had no information on age (N = 936), or on zygosity (N = 140), half-siblings (N = 16), non-biological parents (N = 21), the third person in triplets (N = 28) and spouses of twins (N = 969). Two brothers and two sisters per family were included in the analyses; additional remaining siblings were excluded (N = 39). This resulted in a sample of 12,594 subjects. Tables 1 and 2 give details of the sample composition. Zygosity of same-sex twins was based on DNA polymorphisms or on validated survey questions. For 2660 same-sex twins (997 from complete and 666 twins from incomplete pairs) zygosity information was available from DNA testing.

**Table 1:** Number of participants for genetic analyses.

	Number	Age Men (SD)	Age Women (SD)
Twin 1+2	3077+3335 (6412 twins)	31.44 (11.19)	31.82 (10.97)
	(m: 2036+f: 4376)		
Sister 1+2	902+118 (1020 sisters)		37.26 (11.81)
Brother 1+2	547+55 (602 brothers)	37.70 (13.67)	
Mother + father	2687+1873 (4560 parents)	57.58 (7.21)	53.54 (7.96)

**Table 2:** Number of twins by zygosity.

Twin zygosity	Complete pairs	Total Number of twins
MonoZygotic Males (MZM)	331	970
DiZygotic Males (DZM)	135	529
MonoZygotic Females (MZF)	891	2385
DiZygotic Females (DZF)	358	1165
DiZygotic Opposite Sex (DOS)	355	537/826 (m/f)

The study was approved by the Central Ethics Committee on Research involving human subjects of the Vrije Universiteit Amsterdam, an Institutional Review Board certified by the US Office of Human Research Protections (IRB number IRB-2991 under Federal-wide Assurance-3703; IRB/institute codes, NTR 03-180). All subjects provided written informed consent.

### Phenotype Measures

In survey 7, the screening self-report (CAARS - S:SV) form of Conners' Adult ADHD Rating Scales (CAARS) was included that consists of 30 items accessing ADHD symptom level according to DSM-IV criteria [15] and contains 12 items of the ADHD index. In survey 7 all 30 items were assessed; in survey 8 only the 12 items of the ADHD index were included. The ADHD index is designed to identify the adults in a population that are likely to be diagnosed with ADHD. Each of the 12 items of the ADHD index was scored on the scale from 0 to 4, missing items were handled as per CAARS manual recommendations [15].

### Genetic modeling

Twin-family studies make use of the different degree of genetic relatedness of family members to estimate the relative contribution of genes and environment to the variance of a trait. Monozygotic (MZ) twins are genetically (nearly) identical. Under random mating dizygotic (DZ) twins and non-twin sibling share on average 50% of their segregating genes, while parents and offspring share exactly 50% of their autosomal genes. In quantitative genetic analyses, phenotypic variance is modeled as a function of genetic (G), shared (C), and non-shared environmental (E) influences. Genetic variance can be additive (A), indicating that the effects of alleles are additive, or non-additive (dominance; D) referring to the interaction among alleles. All genetic effects are shared by MZ twins. DZ twins and siblings are correlated 0.5 for their additive genetic values, as are parents and offspring. DZ twins and siblings are correlated 0.25 for their non-additive genetic values, for parents and offspring this correlation is zero. Under non-random mating the correlations among family members for additive genetic values may increase [19]. Twin correlations provide a first impression of the relative contribution of A, C, D and E. The more similar MZ twins are in their phenotypes compared to DZ twins and non-twin siblings, the more variance in a trait is caused by genetic effects. When the DZ correlation is less than half the MZ correlation, there is evidence for D. Differences within MZ twin pairs are due to E which also includes measurement error.

Genetic analyses were carried out using structural equation modeling in Mx [22] using raw-data maximum likelihood estimation of parameters. In a saturated model (with input the 8×8 data matrix of 2 twins, 2 sisters, 2 brothers, mother and father data) we first tested for sex differences in means and variances, for age regression on the ADHD index and for heterogeneity of correlations among family members. The extent to which A, D and E influence the variance in the ADHD index was estimated with the genetic modeling. First, a simple ADE variance decomposition model was fitted to the data, in which the correlation between spouses was simply estimated, but not allowed to influence any other parameters in the model. This would be the case, for example, when living together leads to spousal resemblance. Next, the spousal correlation was modeled as due to phenotypic assortment and several models of familial resemblance were evaluated, allowing for the effects of phenotypic assortative mating. In this last series of models we also evaluated the importance of cultural transmission, in addition to genetic transmission from parents to offspring.

### Statistical testing

The standard approach to the comparison of different models is by means of likelihood-ratio tests, by subtracting the negative log likelihood (-2LL) for a more restricted model from the -2LL for a more general model. This yields a statistic that is distributed as χ^2^ with degrees of freedom (*df*) equal to the difference in the number of parameters in the two models. If the χ^2^-test yields a significant *p*-value (e.g. lower than 0.05 or 0.01), the constrained model is deemed not significantly worse. However, the chi-squared value is inflated as the sample size increases. For this reason, big discrepancies in small samples may not be significant whereas small differences in big sized samples are significant. Given the large sample size in this study (>12,000 subjects) decisions about goodness-of-fit were based on the RMSEA (Root Mean Square Error of Approximation) which is obtained as: RMSEA = √(F0/df), where F0 is the chosen fit function [23]. Good models have an RMSEA of .05 or less. Models whose RMSEA is .10 or more fit poorly.

## Results

Test-retest data were available for 2,519 subjects who took part in NTR surveys 7 and 8. The average age was 41.18 (SD = 13.66) at the first and 45.01 (SD = 13.77) at the second assessments. The correlation between the two assessments was 0.66. There were no differences in average scores for ADHD index (7.75 (SD = 3.79) and 7.54 (SD = 3.87)) between the two assessments. The six month test -retest correlation assessed on 200 unrelated individuals who took part in survey 7 was 0.67.

**Figure 1:**
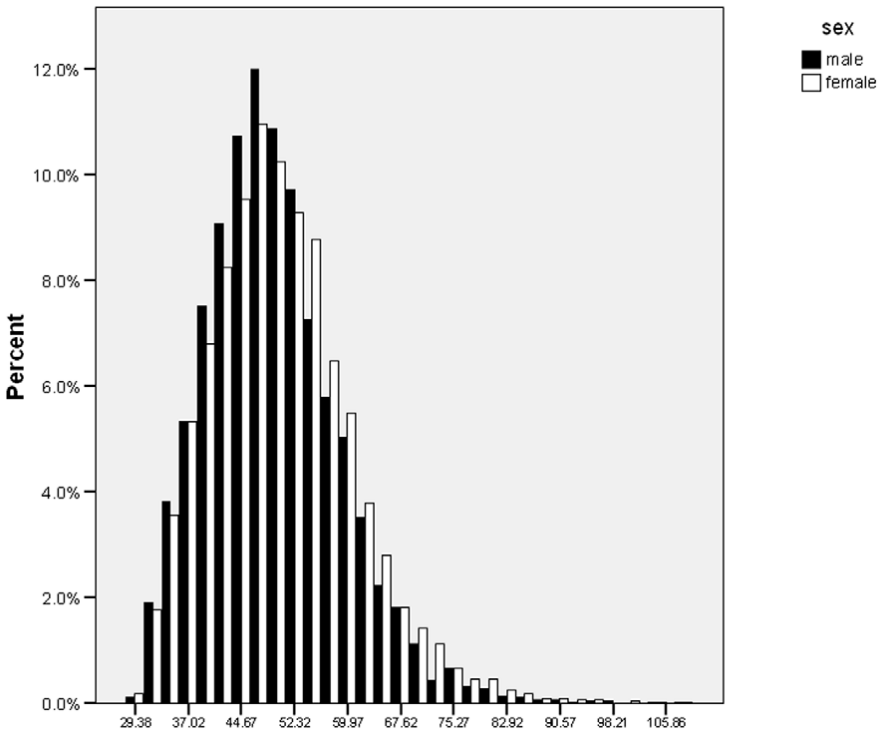
Phenotype distribution of the ADHD index in men and women. ADHD index scores were transformed into T-scores separately by sex.

Figure 1 shows the distribution of the ADHD index in men and women. For this figure, ADHD index scores were transformed into the T-scores within each sex group. A T-score of >65 is considered significant in clinical groups [15]. In the whole sample, 6.8% of women and 7.4% of men had a T-score over 65. In both sexes, the distribution of scores showed a good approximation to a normal distribution with some skewness in the right tail (high end of the scale).

The estimates of means from a saturated model (corrected and uncorrected for age) are given in Table 3 for male and female offspring and for the parents. The estimates of familial correlations are given in Table 4. Model tests are summarized in Table 5. The regression of ADHD index on age (in years) was negative, as expected, and was the same in males and females. The point estimate was −0.02, which is non-significant when judged by RMSEA (<0.05). Women score somewhat higher on the ADHD index than men. There is no generation difference when the regression on age is taken into account. The difference between the sexes is small and the significant chi-squared test is likely to be caused by the very large sample size. Both the sex differences in means and in SD are not significant when judged by RMSEA (<0.05).

**Table 3:** Maximum likelihood estimates for means and standard deviations for ADHD index in offspring and parents and regression estimate on age.

	Mean ADHD (not age corrected)	Mean ADHD (age corrected)	SD ADHD (age corrected)	Age regression
Male offspring	8.07	8.67	3.81	−0.02 (m)
Father	7.57	8.64	3.68	
Female offspring	8.36	9.05	4.11	−0.02 (f)
Mother	7.97	9.12	3.85	

**Table 4:** Maximum likelihood estimates of familial correlations.

	*r ADHD index*	*95% CI*
**MZ twins**	**0.36**	0.31–0.40
MZ Male	0.342	0.244–0.428
MZ Female	0.380	0.324–0.431
**Male first-degree relatives**	**0.09**	0.032–0.158
DZ Male	0.145	−0.092–0.374
brother - male twin	0.081	−0.066–0.221
brother - brother	0.035	−0.202–0.267
father -son	0.076	0.075–0.143
**Female first-degree relatives**	**0.19**	0.151–0.223
DZ Female	0.212	0.112–0.305
sister - female twin	0.217	0.135–0.290
sister - sister	0.107	−0.105–0.303
mother -daughter	0.191	0.144–0.235
**Female-Male first-degree relatives**	**0.12**	0.101–0.156
DZ Opposite Sex	0.112	0.020–0.203
brother - female twin	0.066	−0.049–0.178
sister - brother	0.023	−0.154–0.163
sister – male twin	0.101	0.033–0.216
mother - son	0.122	0.062–0.179
father -daughter	0.139	0.089–0.188
**Parents** (father – mother)	**0.11**	0.061–0.164

**Table 5:** Test results from fitting saturated and genetic models to ADHD data.

Model	NP*	-2LL	Versus model	χ^2^	df	p	RMSEA
1 Saturated model **	27	69807.956	-	-	-	-	-
2 Saturated no age regression	25	69837.384	1	29.428	2	0.0000004	0.0341
3 No sex diff age regression	26	69808.101	1	0.145	1	NS	
**Means and SD**							
4 Means equal within sex	24	69808.291	3	0.190	2	NS	
5 All means equal	23	69825.521	4	17.230	1	0.00003	0.0369
6 SD equal within sex	21	69843.248	5	17.727	2	0.00009	0.0265
7 All SD equal	20	69867.370	6	24.122	1	0.0000009	0.0438
**Equal familial correlations**							
8 in opposite-sex relatives	15	69870.330	7	2.960	5	NS	
9 in male relatives	12	69871.074	8	0.744	3	NS	
10 in female relatives	9	69872.115	9	1.041	3	NS	
11 in all first-degree relatives	7	69881.976	10	9.861	2	0.007	0.0197
12 in MZ twins	6	69882.050	11	0.074	1	NS	
**Variance components**							
13 ADE model	6	69883.032	7	15.662	14	NS	
14 AE model	5	69885.963	13	2.931	1	NS	
**Phenotypic assortment**							
15 ADE & cultural transmission	7	69881.878	7	14.058	13	NS	
16 ADE, no cultural transmission	6	69882.829	15	0.951	1	NS	
17 AE, no cultural transmission	5	69890.142	16	7.313	1	0.0068	0.0240

*NP: Number of estimated parameters.

**Saturated model: 17 familial correlations, 4 means, 4 SD, 2 age regression = 27 parameters; -2LL = -2*Log-Likelihood; df = degrees of freedom for χ^2^ test; NS = not significant; RMSEA = Root Mean Square Error of Approximation.

Table 4 presents the familial correlations for ADHD index in MZ and DZ twins, in sib-sib and twin-sib pairs, in parent-offspring pairs and in parents. All correlations are given conditional on sex. MZM (monozygotic male twin pairs) and MZF (monozygotic female twin pairs) correlations are moderate (0.34 and 0.38, respectively). Correlations in first-degree relatives are somewhat smaller than half the MZ correlations. It is of note that parent-offspring correlations are not lower than DZ or sibling correlations, suggesting that there is little evidence that a different set of genes influences the phenotype in older subjects. For all first-degree relative pairs (male-male, female-female and opposite-sex pairs) correlations could be constrained to be equal and were estimated at 0.09 (male pairs), 0.19 (female pairs) and 0.12 (opposite-sex pairs). The confidence intervals around these correlations overlap and a formal test of equality showed that all correlations between first-degree relatives could be constrained to be the same. The estimate of the correlation in first-degree relatives is 0.14 (95% CI = 0.11–0.16). The correlation in MZM and MZF can also be constrained to be the same and is estimated at 0.36 (95% CI = 0.31–0.40). The correlation between ADHD index scores of parents was estimated at 0.11. We explored if the best explanation for this correlation would be phenotypic assortment or whether spouses who live together for a longer period of time start to increasingly resemble each other. The correlation between the absolute difference in ADHD scores and the mean age of the couple (assuming that older parents have been together for a longer period of time) was 0.005 (N = 1,319).

**Figure 2:**
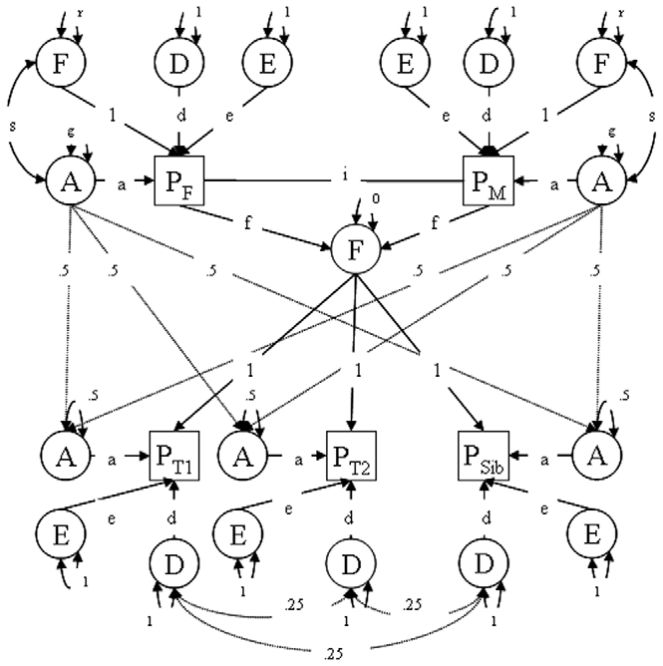
Path diagram of phenotypic assortment model with genetic and cultural transmission from parents to offspring. Squares represent the phenotypes of a DZ twin pair (P_T1_ and P_T2_) with one extra sibling (P_Sib_), and both parents (P_F_ and P_M_). Latent factors are represented by circles and include A (additive genetic factor), D (dominance genetic factor), and E (non-shared environment). F represents vertical cultural transmission whereby the phenotype of the parents influences the environment of their offspring. Assortment of parents is modeled as a copath (i). Simultaneous genetic and cultural inheritance induces a correlation (s) between this environmental factor F and the genetic factor A. Path coefficients a, d and e represent the influence of latent factors on the phenotype. The variance due to vertical cultural transmission is represented by r and the variance of additive genetic factors by g.

The pattern of familial correlations in Table 4 uggests that the familial resemblance that is observed is likely to primarily be explained by genetic factors. A correlation of 0.36 in MZ twins and of 0.14 in first-degree relatives suggests that genetic dominance (non-additivity) may play a role, although dominance would predict lower correlations in parents and their offspring than in siblings and this is not what was seen in the data. Genetic variance components modeling confirmed that the heritability of ADHD index scores is explained by additive genetic factors. In the ADE model, the contributions of A and D to the variance in ADHD index were 29% and 6% respectively. The contribution of D could be constrained at zero and heritability was 33% (95% CI = 0.29–0.36). Next, a model that in addition to genetic also included the cultural transmission paths from parents to offspring was fitted to the twin-family data (Figure 2). This model also included the genetic effects induced by phenotypic assortment specified as a copath [24] which represents an extrinsic correlation that influences the covariance structure of both antecedent and subsequent factors, but does not contribute to their variance and vertical cultural transmission.

Model comparisons (Table 5 model 16 compared to 15) indicated that cultural transmission was not significant. Also, constraining genetic dominance at zero was allowed as judged by the low RMSEA. The final model included an estimate for phenotypic assortment and for additive genetic and unique environmental variance components (4.62 and 10.68, respectively), leading to a heritability estimate of 30%.

## Discussion

This is the first large-scale genetic epidemiology study to report on the heritability of self-rated ADHD symptoms in adults. We assessed ADHD features with the ADHD index, based on the Conners' Adult ADHD Rating Scales in a twin-family sample of over 12,000 adults. Stability of the phenotype was 0.66 both over a 6-month and a 2-year period. When employing a T-score cutoff of >65 the prevalence of adult ADHD was 6.8% in women and 7.4% in men. These prevalence estimates correspond well with those from an epidemiological study using screening and diagnostic assessment of ADHD in 10 countries which reported an overall prevalence of 3.4% and a prevalence for The Netherlands of 5% [25].

The heritability of the ADHD index, when corrected for assortative mating, was estimated to be 30%. There was no difference in heritability between men and women and also not between the parental (average age 55 years) and the offspring generations (average age 34 years). In fact, the parent-offspring correlations were of similar magnitude as the sib-sib and DZ twin correlations, providing evidence that the same set of genes may be playing a role across the adult life-span.

The heritability estimate in adults is substantially lower than the heritability of ADHD and Attention Problems in children. A large longitudinal study of children aged 3–12 year estimated heritability for Attention Problems at 75% at all ages [26] and this is representative of nearly all studies carried out in children [27], [28]. It is unclear if the lower heritability in adults should be explained by age-by-genotype interaction (whereby the expression of the genotype depends on age) or by the fact that ratings of ADHD in children are usually based on parental, teacher or clinician's reports whereas in adults the phenotype is based on self-report.

The age at which heritability decreases from the high estimates in childhood to the lower estimate as reported in the current study may be somewhere during adolescence. A longitudinal study in young adult twins (18–30 years) of self-reported Attention Problems estimated its heritability at 40% [13]. It can be hypothesized that environment during childhood, in so far as it is relevant to ADHD and Attention Problems, is more uniform than later in life, when variation in environment is larger. For example, during childhood a significant amount of time is spend in school by all children. The lower heritability estimate in adults thus may mainly reflect an increase in the contribution of unique environmental influences. The effect of these influences may depend on genotype (genotype-environment interaction). If not explicitly modeled, GE interaction will increase the variance due to unique environment, and thus decrease estimates of heritability [29], [30]. In contrast, the effect of interactions between shared environment and genotype will be included in the genetic variance component. For children there have been reports of interaction between maternal smoking or drinking during pregnancy (by definition an exposure that is shared by twins and likely to be shared among siblings) and offspring genotype [31]–[33]. If effects of such early exposures tend to wane as children grow up, they may also contribute to the higher heritability estimates in children.

The large difference between childhood and adult ADHD heritability is remarkable if we compare these results to those for IQ and cognition. ADHD tends to be associated with a lower IQ [34], but whereas childhood ADHD is highly heritable, childhood IQ shows a rather low heritability (∼25% at age 5) which increases to around 70 to 80% in adults [35]. The developmental patterns of association and whether or not these associations are mediated by genes that influence both IQ and ADHD remain to be investigated.

This study analyzed data from over 12,000 adult subjects. Although the large sample size offered the possibility to test for different models of genetic and cultural inheritance, a limitation of the present study is that the large sample size precluded a clinical diagnosis of the phenotype. Another limitation might be that survey studies in volunteer samples increasingly suffer from non-response. Distel et al. [21] analyzed data from one of the surveys (survey 7) that supplied data for the current analyses and looked at responders from families characterized by a high degree of cooperation (defined as most invited family members taking part in the study) and a low degree of cooperation (defined as one or only a few family members taking part). For genetic (or familial) traits, the data from responders from low-responsive families serve as a proxy for the missing data of their non-responding family members. CAARS data (inattentive and hyperactive/impulsive scales) were included in the analyses. Outcomes tended to be slightly more favorable for individuals from highly cooperative families compared to individuals from less cooperative families, but after correction for multiple testing this effect was not significant for the ADHD scales. These results confirmed those from an earlier study [36] that looked at differences in Attention Problems [12] between subjects from highly and less cooperative families. Non-response bias thus may play a role for ADHD, but its effects are unlikely to be large.

It has been speculated that the heritability of ADHD, especially of the persistent form, may be higher in adulthood than in childhood [37], [38]. This paper signifies that in a general population sample the heritability of ADHD features in adults is substantially lower than that in children. A similar conclusion was drawn when analyzing longitudinal data on Attention Problems in young adults [13]. However, in the current study we could not take into consideration whether or not the adults who took part in the NTR survey studies might have suffered from ADHD in childhood. Currently we are collecting CAARS data in young adult twins who were registered with the Netherlands Twin Register as newborns and for whom parental and teacher data on Attention Problems and Overactive Behavior have been collected from age 3 onwards [27], [39], [40]. This will allow examination of heterogeneity in heritability estimates by stratification of the adult sample into subgroups with persistent and non-persistent ADHD.

This study clearly established the moderate stability and heritability of ADHD in adults. It paves the way for future investigations needed to fully understand the complex architecture of ADHD, such as studies on the heterogeneity of the phenotype, genome-wide linkage and association studies in adults and investigations of the effects of genotype-environment interaction and correlation. As detailed by recent reviews and meta-analyses [4], [37], [41]–[43] attempts to localize and identify genes underlying the vulnerability to ADHD have not yet been very successful. In spite of the lower heritability of adult ADHD as compared to childhood ADHD, we can now attempt to find genetic polymorphisms for ADHD in adults. The NTR-Biobank [44], [45] has collected DNA and RNA samples in over 9500 participants and genotyping is currently under way for a large subsample. Other efforts in adults include the IMpACT initiative [37]. The contribution of (large) numbers of genes across childhood and adulthood can be investigated with genome-wide profiling [46]. In this approach, the effect of multiple SNPs are tested rather than the effects of individual SNPs. These SNPs are not required to reach a genome-wide significance level by themselves, but their combined effect is captured in a genome-wide genetic risk score which can be compared across samples.
